# The impact of bleeding event frequency on health-related quality of life and work productivity outcomes in a European cohort of adults with haemophilia A: insights from the CHESS II study

**DOI:** 10.1186/s13023-023-02690-w

**Published:** 2023-08-03

**Authors:** Lisa Young, Yong Chen, José Alvir, Tom Burke, Enrico Ferri Grazzi, Ian Winburn

**Affiliations:** 1 Pfizer Ltd, Walton-on-the-Hill Tadworth, Surrey, UK; 2 Pfizer Inc, Collegeville, PA USA; 3 Pfizer Inc., New York, NY USA; 4 HCD Economics, Daresbury, Cheshire, UK; 5 Faculty of Health and Social Care, University of Chester, Chester, UK

**Keywords:** Bleeding abnormality, Blood coagulation disorder, Factor VIII, Healthcare expenditures, Socioeconomic factors

## Abstract

**Background:**

Haemophilia A carries a substantial healthcare burden, affecting health-related quality of life (HRQoL). The Cost of Haemophilia in Men: a Socioeconomic Survey II (CHESS II), a retrospective real-world study, characterised the burden of haemophilia and its impact on HRQoL and work productivity. The current analysis explored the impact of bleeding events on HRQoL and work productivity in Europe. This analysis focused on data collected from males aged 18 to 64 years with haemophilia A without inhibitors who were receiving replacement factor products or a monoclonal antibody and were not participating in a clinical trial at the time of study recruitment. Descriptive statistics were analysed using scores from EuroQoL’s EQ-5D-5L index and EQ-VAS analogue scale and the Work Productivity and Activity Index Specific Health Problem (WPAI:SHP) percentage scores stratified by the number of annual bleeding events (ABs) 0, 1, 2, 3–4, or ≥ 5.

**Results:**

Of 918 males with haemophilia A in CHESS II, 318 met inclusion criteria and had data available for HRQoL measures; mean age (SD) was 33.8 (12.1) years and 96% were White. Mean (SD) ABs of 2.7 (2.9) occurred over the preceding 12 months: 20% had 3 or 4 ABs; 17% had ≥ 5 ABs. Mean EQ-5D-5L index scores for patients with 0, 1, 2, 3–4, or ≥ 5 ABs were 0.92, 0.76, 0.76, 0.71, and 0.56, respectively. Mean (SD) EQ-VAS scores were 86.9 (13.6), with 0 ABs versus 69.5 (19.1) for 3 or 4 ABs and 61.2 (17.2) for ≥ 5 ABs. Mean percentage of overall work productivity loss on the WPAI:SHP questionnaire ranged from 9.70 to 0 ABs to 47.65 for ≥ 5 ABs.

**Conclusions:**

In this European sample of adult men with haemophilia A, HRQoL and work productivity scores were lower among those reporting more AB events. Bleeding burden appears to affect HRQoL and productivity; however, this cross-sectional analysis limits the ability to draw firm conclusions on causality.

## Background

Haemophilia A is associated with a substantial healthcare burden, with the greatest burden carried by individuals with severe disease [1]. In 2014, the estimated total annual per-patient expenditures across 5 European countries averaged €199,541 for patients with severe haemophilia A and B, with the majority of the economic burden (98%) driven by drug expenditures [1].

Severe haemophilia is also associated with high rates of both work and school absenteeism due to emergency department and hospital visits, with the rate of absenteeism increasing in line with severity of disease [2]. In an observational study of patients (N = 222) aged 2 to 64 years with haemophilia A (all severities), 34% had at least 1 emergency department visit and 19% had at least 1 hospitalisation, with a mean annual hospital stay of 5.5 days. In adults, there were 14.5 days of absenteeism from school or work due to haemophilia [2].

Globally, 59% of individuals with haemophilia A experience mobility issues, 19% report problems with self-care, 44% have issues with usual activities, 74% have pain or discomfort, and 46% experience anxiety or depression [3]. Individuals with mobility issues (53% vs. 79% with no mobility issues) or with pain or discomfort (31% vs. 84% with no pain/discomfort) were less likely to be employed [3].

Although currently prescribed products are effective for prophylaxis and on-demand treatment, they are associated with a number of limitations, the most serious is the formation of neutralising antibodies (inhibitors) [4–7]. Individuals with haemophilia who develop inhibitors can be effectively managed with recombinant factor VIIa, emicizumab, or activated prothrombin complex concentrate (aPCC) However, these products may present issues in relation to the treatment of specific bleeding events and, in some cases, have limited effectiveness. Additionally, they are costly and greatly increase the economic burden of caring for these patients [4, 8]. Optimal use of factor concentrates may also be limited by difficulty in obtaining reliable venous access, and the cost and frequency of administration of factor replacement [4–7, 9].

Monoclonal antibodies, such as emicizumab, also have limitations as the treatment schedules cannot be adjusted to treat acute bleeding events (these may require factor VIII [FVIII] or bypass therapy) as well as the possibility of injection site reactions in around 20% of individuals [10–12]. Additionally, use of non-replacement therapies in combination with aPCCs may lead to an increased risk of thrombotic microangiopathy and venous thrombosis. Reports of these adverse events have been documented with the use of an aPCC for more than one day and with cumulative doses >100 units/kg/day. Other potential concerns are related to the lack of complete induction of tolerance to FVIII replacement products, and wastage associated with dispensing inaccuracies [13, 14].

There are several treatment gaps in haemophilia A. These include the need to maintain sufficient FVIII levels, improve clinical outcomes, and lower the burden of chronic pain and frequent treatment administration. Products such as rFVIIIFc-VWF-XTEN, [15] siRNA, [16] and monoclonal antibodies targeting tissue factor pathway inhibitor [17, 18] have been in development to reduce the frequency of treatment administration to weekly or monthly injections, respectively, while improving annualized bleeding rates and consequently quality of life. Over the last decade, clinical trials have evaluated gene therapy as a potential therapeutic option for patients with haemophilia [19].

In view of the limitations of the current standard of care for patients with haemophilia A and of the potential transformative nature of gene therapy on the pathways of care, real-world evidence will be crucial in quantifying the substantial burden of disease and to inform the future treatment of this disorder. In order to explore the impact of bleeding events on health-related quality of life (HRQoL) and work productivity in Europe, we conducted an analysis on a subset of adults with haemophilia A without a diagnosis of inhibitors who participated in the Cost of Haemophilia in Men: a Socioeconomic Survey II (CHESS II) retrospective study [20]. The current analysis reviewed data on participants treated with either replacement factor products or a monoclonal antibody but who were not participating in a clinical trial at the time of study recruitment.

## Results

### Patient characteristics

The current analysis focused on the subset of 318 males with mild (18%; n = 57), moderate (27%; n = 85) or severe (55%; n = 176) haemophilia A from the overall CHESS II dataset (N = 1337) who met the inclusion criteria and who had evaluable HRQoL measures. The mean (standard deviation [SD]) age was 33.8 (12.1) years, 96% of patients were White, and 57% were employed (Table 1). Patients were predominantly from Italy (30%) and Spain (26%). Across the sample, 38% (n = 120) of patients received prophylactic treatment and 42% (n = 132) received on-demand treatment.

**Table 1:** Demographics and baseline characteristics of patients with haemophilia A in CHESS II

Patient Characteristics	Number of ABs					
	Total (N = 318)	0 (n = 48)	1 (n = 77)	2 (n = 74)	3/4 (n = 65)	≥ 5 (n = 54)
Age, mean (SD), years	33.8 (12.1)	30.0 (10.4)	33.2 (12.0)	33.5 (12.0)	36.6 (13.1)	35.1 (12.0)
Race/ethnicity, n						
White	305	45	70	71	65	54
Black	7	1	5	1	0	0
Middle Eastern	2	1	1	0	0	0
Asian-Indian subcontinent	4	1	1	2	0	0
Country, n						
France	64	17	16	15	11	5
Germany	24	8	6	8	1	1
Italy	94	8	18	22	21	25
Spain	82	8	22	11	23	18
United Kingdom	54	7	15	18	9	5
Employment Status, n						
Full-time employed	111	24	29	21	22	15
Part-time employed (<30 h/week)	38	4	6	10	12	6
Self-employed	31	4	3	8	10	6
Student	61	9	15	13	13	11
Retired	3	0	2	0	1	0
Unemployed	19	4	3	5	3	4
Homemaker	2	0	2	0	0	0
Physically unable to work due to haemophilia or related complications	14	0	2	2	2	8
Physically unable to work – other reason	1	0	0	0	1	0
Don’t know	38	3	15	15	1	4

AB, annual bleeding event; CHESS II, Cost of Haemophilia in Men: A Socio-Economic Survey II; SD, standard deviation.

### Annual bleeding events

Over the preceding 12 months, a mean of 2.7 (SD 2.9; range 0–26) annual bleeding events (ABs) were reported for the study cohort. Bleeding outcomes worsened with increasing condition severity, with a mean (SD) of 1.1 (0.9), 2.2 (3.4) and 3.5 (2.8) ABs reported for mild, moderate and severe patients, respectively. A total of 15% of patients reported having no ABs over the previous 12-month period; 24% of patients had 1 AB, 23% had 2 ABs, 20% had 3 or 4 ABs, and 17% experienced ≥ 5 ABs over this time period (Table 1). Within the ≥ 5 ABs category, the majority of patients experienced between 5 and 10 ABs, with 7 patients experiencing >10 ABs.

### HRQoL among adults with haemophilia A

**Fig. 1:**
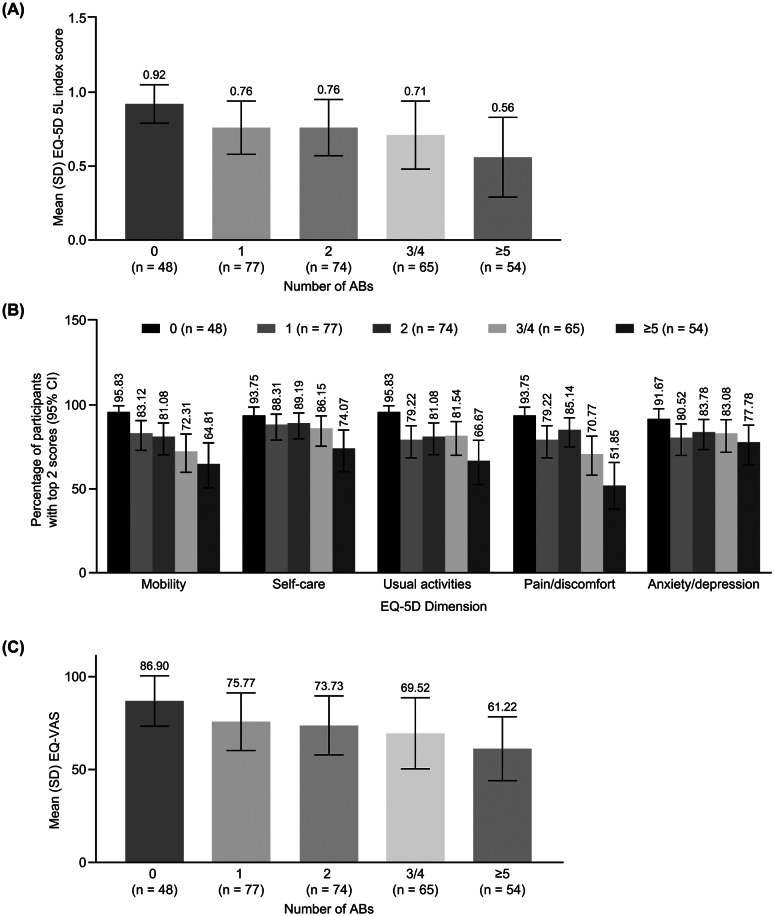
**(A)** Mean EQ-5D-5L scores, **(B)** proportion of patients with top 2 scores for each EQ-5D-5L dimension and **(C)** mean EQ-VAS, stratified by annual bleeding. Mean EQ-5D-5L index score (1 = perfect health with decreasing numbers [negative scores] for the worst health using UK value set and weights). EQ-5D-5L comprises 5 dimensions: mobility, self-care, usual activities, pain/discomfort, and anxiety/depression. Each dimension has 5 levels: (1) no problems, (2) slight problems, (3) moderate problems, (4) severe problems, and (5) extreme problems. The EQ-VAS records the patient’s self-rated health on a vertical visual analogue scale, on which the end points are labelled “the best health you can imagine” and “the worst health you can imagine,” with higher scores representing better health. AB, annual bleeding event; CI, confidence interval; EQ-5D-5L, European Quality of Life 5-Dimension 5-Level; EQ-VAS, European Quality of Life Visual Analog Scale; SD, standard deviation

Mean EQ-5D-5L index scores (1 = perfect health) for patients with 0, 1, 2, 3–4, or ≥ 5 ABs were 0.92, 0.76, 0.76, 0.71, and 0.56, respectively, indicating a decline in overall HRQoL with an increasing number of bleeding events (Fig. 1a). The percentage of patients in the top 2 levels of the EQ-5D-5L domain scores reflects a trend toward reduced HRQoL with an increasing number of ABs (Fig. 1b). The majority of patients with no ABs (96%) reported no/slight mobility problems compared with 72% of those with 3 or 4 ABs and 65% of patients with ≥ 5 ABs. Similarly, more than 90% of patients with no ABs versus those with ≥ 5 ABs reported no/slight problems with self-care (94% vs. 74%), usual activities (96% vs. 67%), no/slight pain or discomfort (94% vs. 52%), and no/slight anxiety/depression (92% vs. 78%).

Mean (SD) EQ-VAS scores, which reflect patients’ self-rated health, were 86.9 (13.6) for patients with 0 ABs compared with 69.5 (19.1) for those with 3 or 4 ABs and 61.2 (17.2) for those with ≥ 5 ABs, which indicates that poorer self-rated health was reported by patients with a greater number of ABs (Fig. 1c).

### Work productivity loss

**Fig. 2:**
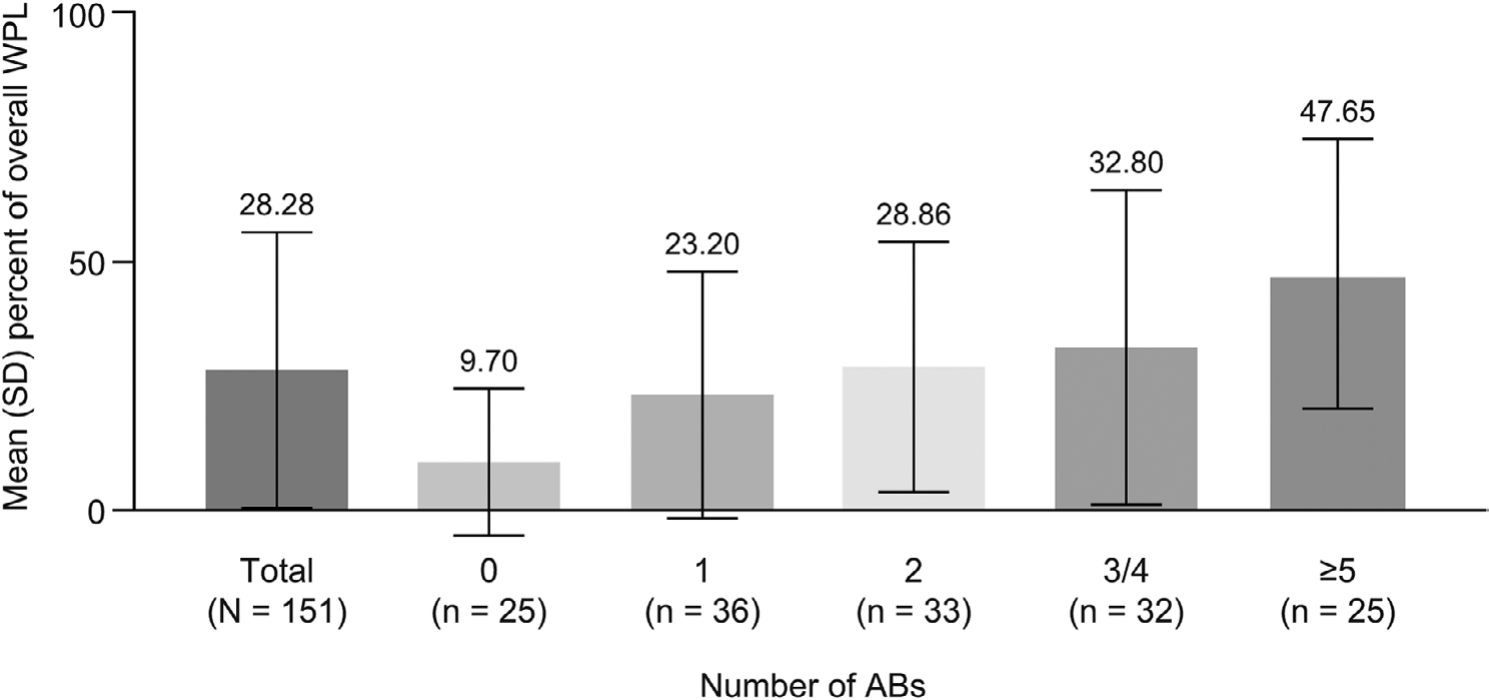
Percentages of overall work productivity loss due to haemophilia A. All data assessed are from the CHESS II study. AB, annual bleeding event; SD, standard deviation; WPL, work productivity loss

Of 318 patients included in the current study, 180 (57%) were employed and were invited to provide information on work productivity related to haemophilia via the Patient Public Involvement Engagement (PPIE) questionnaire. Of the 180 participants, 171 were in paid employment and eligible to provide work productivity loss (WPL) information. Of these 171 participants, 151 reported all items necessary to assess their WPL via the Work Productivity and Activity Impairment: Specific Health Problems (WPAI:SHP) Questionnaire (included in the PPIE questionnaire) and were included in the WPL analysis. The percentages of overall WPL increased with the number of ABs (mean ranged from 9.70 for patients with 0 ABs to 47.65 for those with ≥ 5 ABs) (Fig. 2).

## Discussion

This analysis of a subset of patients in a retrospective real-world study describes the burden of illness in patients with haemophilia A across 5 European countries. The results indicated that a significant HRQoL burden is associated with bleeding events among those with haemophilia A.

The results of this analysis highlighted a substantial burden imposed on patients by haemophilia A both in terms of HRQoL and work productivity. The greatest impairments in WPL and across all individual EQ-5D-5L domains were reported in individuals with a higher number of ABs. These findings were aligned with the patients’ subjective self-rated health assessments, reflecting poorer health in those who experienced a greater number of ABs. The results of the current study are consistent with previous analyses that showed a significant burden of disease in patients with haemophilia, with lower scores observed on the mobility, self-care, pain/discomfort, and anxiety/depression domains [3]. In addition, as in the current study, previous analyses reported reduced HRQoL associated with a greater frequency of ABs [3].

The results of our analysis highlighted that increasing levels of bleeding events were associated with increased WPL, with the largest loss observed in those with 3 or more ABs. The high burden of indirect expenditures associated with haemophilia A, including loss of productivity in the form of missed work time/absenteeism, is reported in previous analyses with similar proportions of patients in employment as in the current analyses (47.3–60%) [2, 3, 21, 22]. In these analyses, up to 80% of patients reported an adverse effect on their employment [2, 3, 21, 22]. Lower employment was associated with a higher reported bleeding event frequency and with lower HRQoL. In addition, as in the current study, previous analyses reported lower employment was associated with increased disability and pain [3]. Although an analysis of indirect expenditures was beyond the scope of the current analysis, WPL accounts for the majority of indirect cost for haemophilia, with a reported annual indirect cost previously estimated to be approximately €6,075 per patient with haemophilia (based on 2017 data) [1].

The findings of this analysis should be interpreted in light of some limitations. This was a retrospective analysis of existing observational data from CHESS II, composed of data from patients’ medical records as reported by their treating physicians as well as questionnaires completed by the patients in select European countries. Due to the retrospective, cross-sectional, and physician- and patient-reported nature of the CHESS II study, a degree of selection and recall bias, as well as potential for data extraction errors cannot be excluded. Finally, this analysis included adult patients up to age 64 years but did not include a subgroup analysis by age group; it would be of interest to explore the impact of age on the findings, as quality of life and work productivity have been shown to decrease in older persons with haemophilia [23].

This analysis provides useful insight on the burden of the intensity of bleeding in a European cohort of patients with haemophilia A of any severity, also highlighting an important unmet need in the management of this patient population. These data from routine clinical practice suggest improvements in the treatment of haemophilia A may result in better overall management and improved bleed-related outcomes. The findings of this analysis also suggest that further investigation of other aspects of replacement therapy (i.e., type of treatment, dosage, frequency of injections) and sequelae of bleeding (i.e., joint damage or chronic pain) and their effects on HRQoL may be beneficial to condition management. Management of patients with haemophilia A is further complicated by difficulties in obtaining reliable venous access and the requirement for frequent infusions and, therefore, continues to present a challenge for clinicians [5]. Against this background, the promise of gene therapy represents a potential transformation in the current treatment paradigm for eligible people with haemophilia A.

## Conclusions

The CHESS II study showed that severe haemophilia A is associated with reduced HRQoL and reduced work productivity, which worsens with a greater number of ABs. Treatment modalities that offer the potential for reduced ABs, along with consequent improvements in HRQoL and work productivity, may offer renewed hope for people with haemophilia A.

## Methods

### Study design and population

We report the findings of an analysis of the CHESS II data in a subgroup of patients with haemophilia A reporting sufficient data from both EQ-5D-5L and work productivity from 5 European countries (i.e., France, Germany, Italy, Spain, and the United Kingdom). The design and methodology of the CHESS II study have been reported previously [1]. Briefly, CHESS II was a cross-sectional retrospective study of adult males with inherited haemophilia A or B of any severity with or without inhibitors. The overall study took place across 8 countries in Europe (Denmark, France, Germany, Italy, Netherlands, Romania, Spain, and United Kingdom) between November 2018 and November 2020. Key eligibility criteria for inclusion in the current analysis were adult males aged 18 to 64 years enrolled in the CHESS II study with a diagnosis of hereditary haemophilia A of any severity in the 12 months prior to the index date.

Key exclusion criteria for the current analysis included enrolment in clinical trials currently or within the last year, a diagnosis of inhibitor, a diagnosis of other haemophilia subtypes, and/or bleeding disorders.

### CHESS II study assessments

Eligible physicians retrospectively enrolled, on average, 7 to 8 patients with haemophilia for a 12-month period prior to the clinical consultation (index date). Physicians completed an electronic case report form for the patients they most recently consulted with. Bleeding events in the last 12 months were recorded.

After clinical consultation, enrolled patients were invited to complete a corresponding printed PPIE questionnaire regarding demographic and clinical characteristics, a self-reported HRQoL, and work participation and productivity, among other haemophilia-related patient-reported outcomes.

HRQoL was evaluated using the EQ-5D-5L questionnaire and EQ-VAS [24]. The EQ-5D-5L comprises 5 domains, including mobility, self-care, usual activities, pain/discomfort, and anxiety/depression. Based on the mean EQ-5D-5L index score, a score of 1 equates to perfect health, with decreasing numbers (negative scores) indicating incrementally worse health, using a UK value set and weights. The EQ-VAS recorded each patient’s self-rated health on a scale from 100 (the best health you can imagine) to 0 (the worst health you can imagine).

WPL was measured using the WPAI:SHP questionnaire [25]. It is a validated, widely used instrument for measuring the impact of a condition on an individual’s work and is, in turn, a helpful tool for estimating the indirect costs of haemophilia and burden on society [26–28].

Sociodemographic data were also collected along with disease history, including information on haemophilia-related symptoms, treatments, and consultation history, as well as history of factor replacement usage, history of treatment regimens, and use of factor replacement in the 12 months prior to the index date. Data on societal and indirect expenditures were collected over the 12-month period prior to the index date.

### Data analysis

Data were summarised using descriptive statistics. Mean and SD were used to summarise continuous variables, and frequency and percentages were used to *s*ummarise categorical variables. SAS version 9.4 or later was utilised for all data analyses. Patients were stratified according to number of ABs. After excluding patients with missing values for HRQoL measures from further analysis, EQ-5D-5L domain scores and overall scores were stratified by annualised bleeding events. Results on WPL are expressed as a percentage score, and the mean percentage impairment score was reported across bleeding outcomes.

## Data Availability

The dataset that supports the conclusions of this study may be available from HCD Economics, Ltd, but restrictions apply to the availability of these data, which were used under license for the current study, and are not publicly available. Data may be available from the authors upon reasonable request and with permission of HCD Economics Ltd.

## Abbreviations

AB: Annual bleeding event
CHESS II: Cost of Haemophilia in Men:A Socioeconomic Survey II
CI: Confidence interval
EQ-5D-5L: EuroQoL 5-Dimension 5-Level
EQ-VAS: European Quality of Life Visual Analog Scale
FVIII: Factor VIII
HRQoL: Health-related quality of life
PPIE: Patient Public Involvement Engagement
SD: Standard deviation
WPAI:SHP: Work Productivity and Activity Index Specific Health Problem
WPL: Work productivity loss
